# Co-existence of hepatocellular carcinoma and cystic echinococcosis

**DOI:** 10.1186/s13027-020-0275-0

**Published:** 2020-01-29

**Authors:** Ran Bo, Aimaiti Yasen, Yingmei Shao, Wenbao Zhang, Renyong Lin, Tiemin Jiang, Hao Wen, Hui Xiao, Tuerganaili Aji

**Affiliations:** 1 Department of Liver Hydatid Disease, Digestive and Vascular Surgery Center, The First Affiliated Hospital of Xinjiang Medical University, Urumqi, 830011 Xinjiang Uyghur Autonomous Region People’s Republic of China; 2 School of Public Health, Xinjiang Medical University, Urumqi, 830011 Xinjiang Uyghur Autonomous Region People’s Republic of China; 3 State Key Laboratory on Pathogenesis, Prevention and Treatment of High Incidence Diseases in Central Asia, Xinjiang Medical University, Urumqi, 830011 Xinjiang Uyghur Autonomous Region People’s Republic of China; 4 Xinjiang Key Laboratory of Echinococcosis, The First Affiliated Hospital of Xinjiang Medical University, Urumqi, 830011 Xinjiang Uyghur Autonomous Region People’s Republic of China; 5 Clinical Medical Research Institute, The First Affiliated Hospital of Xinjiang Medical University, Urumqi, 830011 Xinjiang Uyghur Autonomous Region People’s Republic of China

**Keywords:** Hepatocellular carcinoma, Cystic echinococcus, Co-existence, Survival time

## Abstract

**Purpose:**

Co-existence of hepatocellular carcinoma (HCC) and cystic echinococcus (CE) is extremely rare. *Echinococcus granulosus* may exhibit a protective effect against cancer. Herein, this study aimed to evaluate the possible effects of echinococcal infection on HCC patients.

**Methods:**

Three thousand three hundred hepatic CE patients and 815 HCC patients were retrospectively reviewed between January 2010 and December 2018 in Xinjiang, China, and these patients were 1:5 matched according to their sex, age and tumor TMN stage, and only 13 patients coexisted both CE and HCC. Preoperative ultrasonography (US), computed tomography (CT), liver magnetic resonance imaging (MRI) and dot immune-gold filtration assay (DIGFA) were used for preoperative identification and intraoperative specimens from liver resections were pathologically examined for further confirmation. Survival time was analyzed through Cox proportional hazard model analysis.

**Results:**

The co-existing incidence rate of two diseases was 0.39%. For these concurrent cases, HCC was all at the advanced stage and CE lesions were inactive. Median survival time for HCC patients was 6 month (1–17). However, it was 8 month (3–90) for the co-existing cases and was much longer than the median survival time of HCC patients (*P*<0.05), which was closely associated with tumor size, location, TMN stage and hydatid size, location, classification. Four of the patients underwent surgical intervention and their median survival time was 17 month (3–68).

**Conclusions:**

*Echinococcus granulosus* may elicit a protective effect against the development and progression of HCC, while more basic and clinical researches are needed.

## Introduction

Hepatocellular carcinoma (HCC) is a leading cause of cancer-related mortality throughout the world, with the sixth highest cancer incidence and the fourth highest cancer mortality in 2015 [1]. Despite continuous improvement in both diagnosis and treatment, prognosis for HCC patients is still poor because they are often diagnosed at symptomatic and advanced stages, and the treatments such as surgical resection, liver transplantation, or radio-frequency ablation for these stages are usually limited [2].

Cystic hydatid disease or cystic echinococcosis (CE) is a globally endemic zoonosis caused by the larval cyst stage of the dog tapeworm e*chinococcus granulosus*. The disease seriously impacts both public health and animal production in Central Asia, the Mediterranean countries, and South America [3]. The most target organ for CE is liver, followed by lung, brain and other organs [4]. Surgical removal of cyst is the curative treatment and oral taking albendazole is alternative treatment, but it may take for long time up to years [5].

Concomitant presence of CE and HCC is a fairly rare clinical scenario, while growing studies have shown that echinococcosis is closely associated with the occurrence and progression of various malignant tumors. Moreover, it has been reported in a large retrospective study that e*chinococcus granulosus* infection results in a significantly lower prevalence of cancer in CE patients [6]*.* Meanwhile, certain parasite antigens may inhibit tumor growth. Thus, e*chinococcus granulosus* may exhibit a protective effect against cancer [7]*.* However, whether echinococcal infection could provide a possible approach for cancer therapy is still unclear.

In this study, we retrospectively analyzed the clinical data of patients with concomitant CE and HCC and the corresponding HCC patients in our hospital (the First Affiliated Hospital of Xinjiang Medical University). It was showed that echinococcal infection may prolong the survival time of HCC patients.

## Methods and materials

### Patient population

From January 2010 to December 2018, 3300 hepatic CE patients underwent surgery for removal of liver hydatid cysts at our hospital. Among them, only 13 cases coexisted both conditions (CE and HCC). Besides, we also reviewed 815 HCC patients to compare their survival time with the co-existing conditions, and we 1:5 matched the concomitant patients with the corresponding HCC patients according to their sex, year and tumor TMN (T: tumor, N: node, M:metastasis) stage. Detailed information of the patients was shown in Table 1.

**Table 1:** Demographic characteristics and clinical data of 13 patients with concomitant HCC and CE

No	Sex/age	HCC			CE		Classification of concomitant HCC and CE	
		Location	Size (cm)	TNM stage	Location	Size (cm)	Classification	lesions
1	F/38	Right lobe	11.00	IV A (T4N1M0)	Left lobe	6.00	CE4/T4D6C0	Type 3a HCC/CE lesion
2	M/82	Left lateral, right anterior lobe	3.75	III A (T3aN0M0)	Left medial lobe	4.18	CE4/T4D4C0	Type 3b mHCC/CE lesion
3	M/49	Right lobe	4.13	III B (T3bN0M0)	Left lobe	4.00	CE4/T4D4C0	Type 3a HCC/CE lesion
4	M/67	Right lobe	10.08	IV B (T3bN1M1)	Left lobe	15.60	CE4/T4D15C0	Type 3a HCC/CE lesion
5	M/78	Right lobe	10.00	III B (T3bN0M0)	Right posterior lobe	14.00	CE4/T4D14C0	Type 1b HCC/CE lesion
6	F/67	Right posterior lobe	3.41	III B (T3bN0M0)	Right lobe	3.26	CE5/T5D3C0	Type 1b HCC/CE lesion
7	F/27	Left lobe	6.80	III C (T4N0M0)	Right lobe lobe	16.00	CE4/T2D16C0	Type 4a HCC/CE lesion
8	M/82	Right lobe	9.20	III C (T4N0M0)	Right posterior lobe	7.69	CE5/T5D7Cb	Type 1b HCC/CE lesion
9	M/44	Diffused	2.50	III B (T3bN0M0)	Right lobe	9.20	CE4T4D9Cb	Type 4b mHCC/CE lesion
10	F/62	Right lobe	15.00	IV B (T3aN0M1)	Left lobe	9.27	CE4T4D9Cb	Type 3a HCC/CE lesion
11	M/59	Left lobe	7.70	IVA (T4N1M0)	Right lobe	6.30	CE4T4D6Cb	Type 4a HCC/CE lesion
12	F/41	Right lobe	7.40	IV B (T3bN1M1)	Right lobe	1.10	CE4/T4D1C0	Type 1b HCC/CE lesion
13	M/67	Right lobe	12.8	IV A (T4N1M0)	Left lobe	9.00	CE2/T2D9C0	Type 3a HCC/CE lesion

### Preoperative assessment

**Fig. 1:**
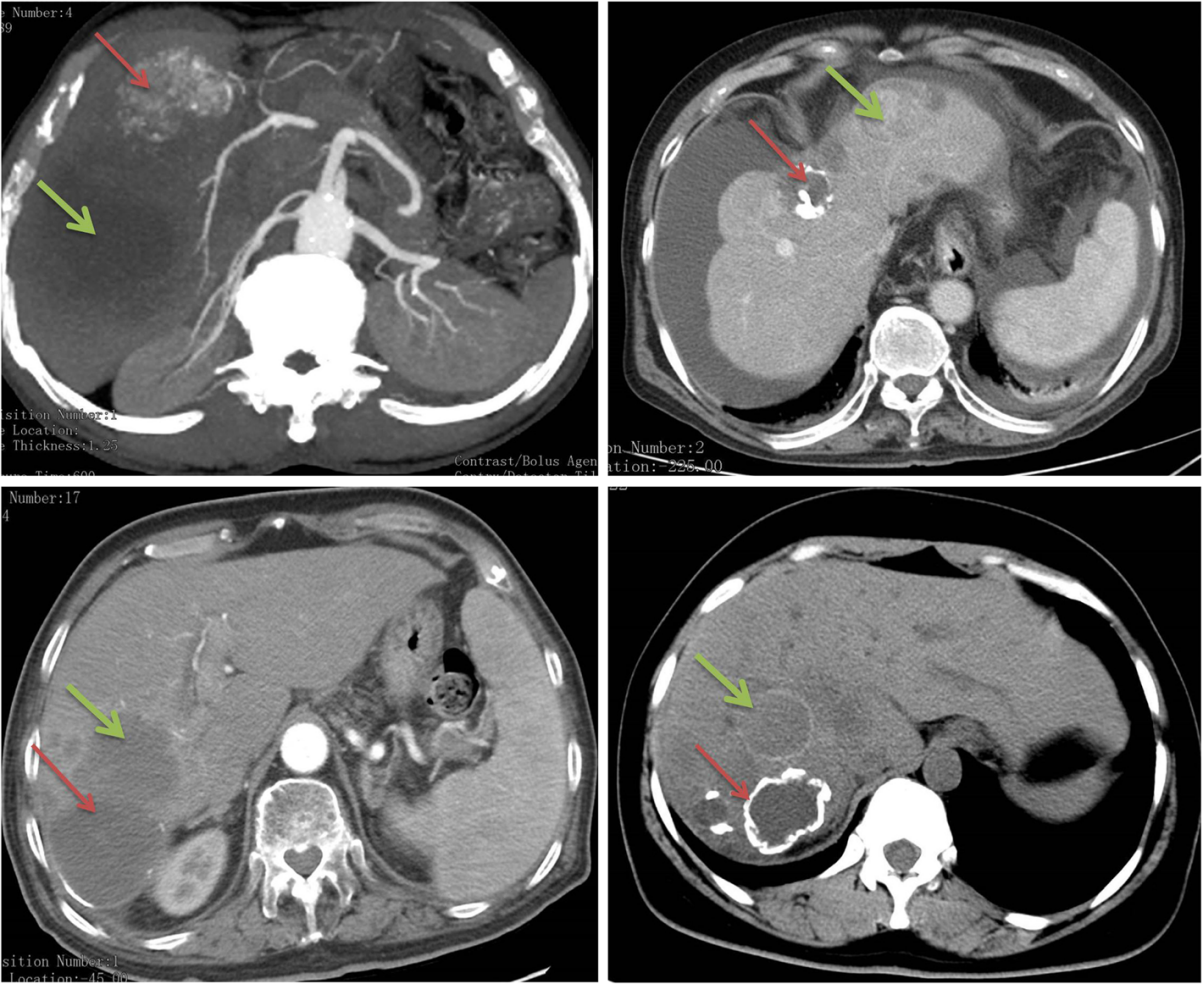
Representative imaging. Red arrow represents hepatic CE lesions; Green arrow represents HCC lesions

Preoperative computed tomography (CT), liver magnetic resonance imaging (MRI), Ultrasonography (US) were systematically used to assess liver lesion’s size, location, parenchymal, vascular as well as biliary extension and extra-hepatic metastasis of the lesions (Fig. 1). The dot immunogold filtration assay (DIGFA) was used to detect the serum echinococcosis specific antibodies [8, 9]. TMN staging of HCC was categorized according to American Joint Committee on Cancer [10] and hepatic CE was categorized according to World Health Organization Informal Working Group on Echinococcosis (WHO-IWGE) PNM (P: parasite mass in the liver, N: involvement of neighboring organs, M: distance metastasis) classification system [11]. In addition, Romic classification system was used for the classification of concomitant cases [12].

### Post-operative management and follow-up

Post-operative complications were assessed based on the Clavien Classification [13]. All subjects undergoing operation received standard postoperative albendazole treatment for at least two years [6]. Patients’ follow-up data were collected through outpatient review and/or telephone. The deadline for follow-up was December 2018. The overall survival time of these patients referred to the period from definite diagnosis to patients’ death time, and patients who were lost follow-up or died due to other accidental reasons were excluded from this study.

### Statistics

Results were shown as median value with range, and Student t-test was used for quantitative data when necessary. χ^2^ test or Fisher’s exact test where number was less than 5 was used for the analysis of qualitative data. *P*<0.05 was considered statistically significant.

## Results

### Study cohort

From January 2010 to December 2018, 3300 CE patients were treated in the First Affiliated Hospital of Xinjiang Medical University. Among them, 13 cases coexisted with HCC and CE, and the co-occurrence rate was 0.39% (13/3300). There were 8 male and 5 female patients, accounting for 61.54% (8/13) and 38.46% (5/13) respectively. The male patients had a median age of 67 (44–82), and female patients had a median age of 41 (27–67). The median age of co-existing patients was 62 years (27–82). Table 1 shows the basic data of the patients.

### Characteristics of HCC and hydatid cyst

**Fig. 2:**
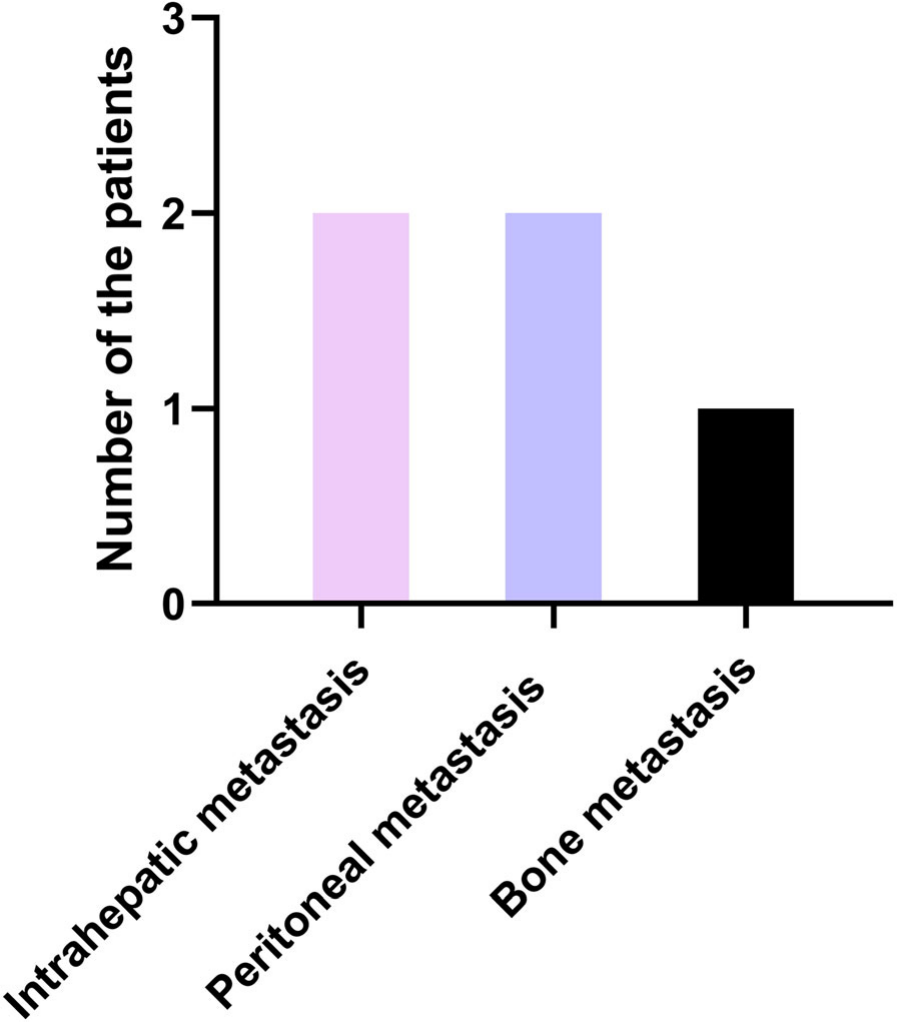
Frequency of HCC metastasis among CE patients. There occurred Five cases with distant metastases including two with intrahepatic multiple metastasis, two with peritoneal metastasis and one with bone metastasis

Among the 13 patients, 9 cases (69.23%) had their tumor lesions located in the right lobe and the median size of tumor was 7.98 cm (ranged from 2.5 cm to 12.8 cm). Most of the HCC patients were at advanced stage (III-IV) according to AJCC system. Among them, 7 cases (53.85%) were in stage III and 6 cases (46.15%) were in stage IV. Five cases (35.46%) were accompanied by distant metastasis including two with intrahepatic multiple metastasis, two with peritoneal metastasis and one with bone metastasis, which was presented in Fig. 2. The median size of echinococcal cysts was 8.05 cm (ranged from 1.1 cm to 15.6 cm), with 92.31% (12/13) of the cysts being classified as inactive cysts (CE4 and CE5). The specific classification of concomitant HCC and CE lesions were categorized according to Romic classification proposal, which is mainly based on the anatomical location of CE and HCC lesions. Type 3a HCC/hydatid lesion in five patients and type 1b HCC/hydatid lesion in four patients were seen respectively. HCC lesion was located in the right anterior lobe and further extended to the left lobe in one patient, while the CE lesion was located in the left lobe without extension, which was categorized as type 3b mHCC/hydatid lesion. Comparatively, HCC lesion was initially located in the left lobe and broadly extended to the other lobes in another patient, and the CE lesion was in the right lobe, which then categorized as type 4b mHCC/hydatid lesion. In addition, type 4a HCC/hydatid lesion was found in one patient. Most importantly, all of these CE and HCC lesions in the liver were present as separated.

### Diagnosis of co-existing condition and treatment methods

Routine laboratory tests including liver function, tumor markers were unremarkable, which were presented in Table 2. In this study, 8 patients received four golden hydatid tests. Serological DIGFA showed that 1 case was strong positive and 2 cases were weakly positive. However, 5 cases were serologically negative against these antigens. Preoperative CT or MRI, US were essential to initially diagnose patients with both conditions (HCC and CE) and representative imaging results were shown in Fig. 1. The intraoperative specimens from the liver resections were pathologically examined in the patients undergoing surgery. After careful assessment by a multidisciplinary team (MDT), including hepatobiliary surgeons, hepatologists, interventional therapists, radiologists and anesthesiologists, subtotal peri-cystectomy and partial hepatectomy was performed in two patients. Another two patients received subtotal peri-cystectomy, partial hepatectomy and cholecystectomy. In addition, chemoembolization (Pirarubicin hydrochloric acid 30 mg + Oxaliplatin 100 mg) through hepatic artery was performed in one patient. However, conservative supporting care was given for eight patients due to the advanced stage of HCC lesions. Antihelmintic thereapy (oral albendazole at an average dozage of 15 mg/kg/day) was administrated to all patients.

**Table 2:** Main clinical, laboratory test results and treatment methods of the patients

No	Liver function				Tumor markers				Echinococcosis antigens				Treatment method
	ALT	AST	ALB	TDIL	AFP	CEA	CA19–9	CA125	EgCF	Egp	EgB	Em2	
1	148.50	118.60	26.40	58.50	59.71	1.94	5.87	464.20	None				Conservative treatment
2	79.30	64.69	34.70	3.45	4.20	1.55	4.68	600.00	None				Conservative treatment
3	221.90	239.71	31.20	20.65	1000.00	2.06	49.18	21.50	None				Conservative treatment
4	46.60	25.11	34.60	14.20	1000.00	11.34	7.09	297.40	None				Conservative treatment
5	25.60	20.60	30.61	11.51	0.24	0.60	8.43	44.08	(+)	(±)	(+)	(±)	Subtotal peri-cystectomy + partial hepatectomy+ cholecystectomy
6	22.82	26.25	36.14	38.41	60.34	10.54	7.98	200.75	None				Chemoembolization with hepatic arteriography
7	34.70	33.30	19.80	25.05	1000.00	50.54	29.45	400.23	(++)	(++)	(++)	(+)	Subtotal peri-cystectomy+ partial hepatectomy+ cholecystectomy
8	45.10	42.80	29.10	13.20	2.20	2.23	293.61	34.70	(−)	(−)	(−)	(−)	Subtotal peri-cystectomy+ partial hepatectomy
9	37.40	23.40	43.70	29.70	7.50	0.56	2.63	17.10	(−)	(−)	(−)	(−)	Subtotal peri-cystectomy + partial hepatectomy
10	1395.00	305.00	21.00	38.80	6.45	5.34	89.95	400.23	(−)	(−)	(−)	(−)	Conservative treatment
11	158.60	78.90	18.10	49.65	237.24	3.69	319.97	204.98	(−)	(−)	(−)	(−)	Conservative treatment
12	1148.60	271.42	36.70	60.60	138.00	101.80	4.82	111.30	(−)	(−)	(−)	(−)	Conservative treatment
13	1.93	102.00	21.00	209.20	208.45	78.93	57.24	130.45	(+)	(+)	(−)	(−)	Conservative treatment

### Distribution of etiological factors

These 13 concomitant HCC and CE cases were matched with the corresponding HCC patients. The distributions of etiological factors between co-occurrence cases of HCC and CE as well as their matched controls were shown in Table 3. As anticipated, there were no significant differences in the etiological factors between groups (*P*>0.05). There were no subjects with the history of aflatoxin exposure and hemochromatosis in both groups. In patients with coexisting HCC and CE, virus infection, including hepatitis A, B and C virus, was present in seven cases and cirrhosis was present in ten cases, which were slight higher than that in the corresponding HCC patients. However, HCC patients were more likely to consume alcohol and to have the medical history of nonalcoholic steatohepatitis (NASH). In addition, HCC patients also tended to have a family history of cancer.

**Table 3:** Description of study variables in patients with co-occurrence of HCC and CE (HCC + CE) as well as control patients with HCC (HCC)

Condition	Variable	HCC + CE (*n* = 13) No. (%)	HCC (*n* = 65) No. (%)	^a^*P*
Gender	Male	8 (61.54)	40 (61.54)	1.00
	Female	5 (38.46)	25 (61.54)	
Virus infection	No	6 (46.15)	31 (47.69)	0.7769
Hepatitis A, B and C virus	Yes	7 (53.85)	34 (52.31)	
Alcohol consumption	No	13 (100)	63 (96.92)	0.0810
	^*^Yes	0 (0)	2 (3.08)	
Aflatoxin	No	13 (100)	65 (100)	1.00
	Yes	0 (0)	0 (0)	
NASH	No	13 (100)	63 (96.92)	0.0810
	Yes	0 (0)	2 (3.08)	
Cirrhosis	No	3 (23.08)	17 (26.15)	0.6218
	Yes	10 (76.92)	48 (73.85)	
Hemochromatosis	No	13 (100)	65 (100)	1.00
	Yes	0 (0)	0 (0)	
Family cancer history	No	12 (92.31)	59 (90.77)	0.7998
	Yes	1 (7.69)	15 (9.23)	

^*^ ≥  1 glasses weekly for the past 6 months; *NASH* nonalcoholic steatohepatitis; ^a^*P*-Value from χ^2^ test or Fisher‘s exact test where number is less than 5

### Survival analysis of relevant factors

**Fig. 3:**
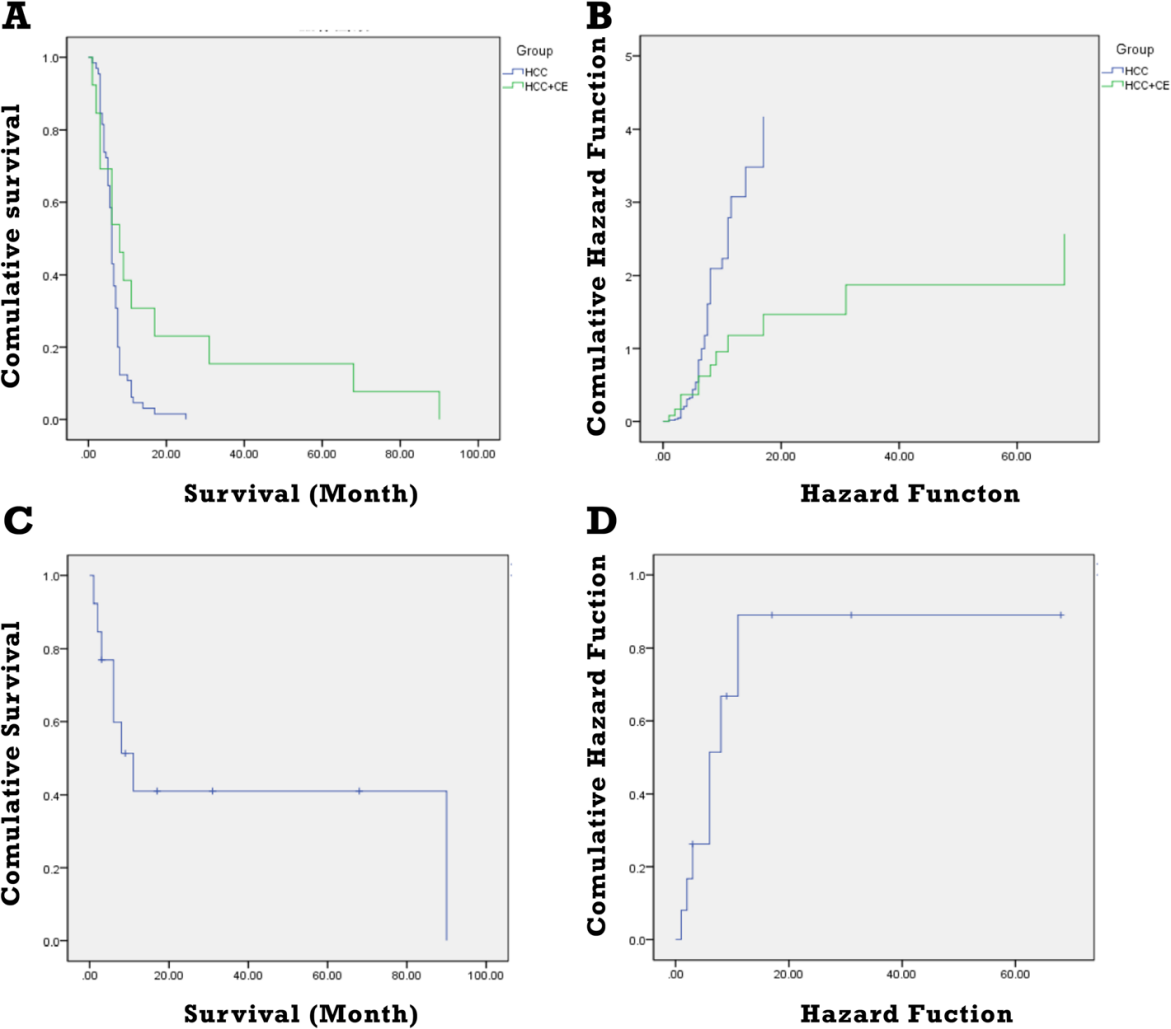
Survival time in HCC patients and co-existing patients. **a**: Comparison of survival time in HCC patients and co-existing patients. **b**: Comparison of hazard function in HCC patients and co-existing patients. **c**: Survival curve in co-existing patients. **d**: Hazard function in co-existing patients

In order to compare the survival time of co-existing patients and HCC patients, 815 HCC patients were also reviewed. Median survival time for HCC patients was 6 month (1–17). However, that was 8 month (3–90) in the co-existing patients and was much longer than the median survival time of HCC patients (*P*<0.05), which was closely associated with tumor size, location, TMN stage and hydatid size, location, classification. Fig. 3 shows the survival time of these patients. Among the concurrent patients, four underwent surgical intervention and their median survival time was 17 month (3–68), suggesting that echinococcal infection may prolong the survival time of HCC patients. Therefore, surgical intervention and post-operative comprehensive treatment are recommended for patients with concurrence of HCC and CE.

## Discussion

Co-existence of HCC with echinococcosis is rare and closely associated with patients diminished life quality and significant morbidity. Recent studies in Europe and central Asia have shown that there may exist a connection between hydatids and tumors [5, 14, 15]. In this study, among 3300 liver CE patients only 13 cases (0.39%) had HCC, whose lower percentage was consistent with the previous findings. A large retrospective study has shown that among the HCC patients only two of them were accompanied by echinococcosis [16]. Moreover, researchers have retrospectively analyzed 1200 patients with various hematological diseases, who received treatment in Turkey hospitals from 1985 to 2003, and demonstrated that there only occurred co-existence of acute leukemia and hepatic hydatid disease in one case [15].

In these concurrent cases, all HCC patients were at the advanced stage. Seven patients were at stage III and six patients were at stage IV. Five cases were accompanied by distant metastasis (38.46%). Among them, two cases were with intrahepatic metastasis, two cases with peritoneal metastasis and one case with bone metastasis. Possible reasons for this above clinical picture may be as follows: (a) hepatic hydatid disease is usually distributed in pastoral area and most patients seek treatment until they have significant clinical symptoms due to their poor living conditions; (b) tumor lesions may be overlapped by the CE lesions in some early stage HCC patients.

Inactive hydatid lesion, including CE4 and CE5, account for the majority of co-existing cases. In this current study, 10 cases were categorized as CE4, 2 cases as CE5. Positive rate of DIGFA was relatively low. 8 patients received four golden hydatid tests in our study, among which 1 case was strong positive and 2 cases were weakly positive. Positive rate for this examination was far below the reported average level by some researchers that positive rate could be up to 80% in most cases [16, 17]. This phenomenon may be explained by the fact that hepatic echinococcosis is always at the senescence stage when patients seek treatment and diagnostic accuracy of the reported hydatid test may be closely associated with echinococcosis viability.

Preoperative diagnostic rate for patients with concomitant HCC and CE was far from satisfactory. In this study, four patients underwent surgical intervention, and liver hydatid had ruputerd into biliary tract when one patient was preoperatively diagnosed, whose surgical pathology confirmed as CE co-existing with cholangiocarcinoma. The preoperative diagnosis for two cases was liver cyst hydatid with infection, whose surgical pathology confirmed as CE co-existing with hepatic carcinoma. However, only one patient was precisely diagnosed before operation through general imaging examination. Both CE and HCC are chronic disease with no typical clinical manifestations, while CE lesions can exhibit characteristics of tumour-like, infiltrative growth in the liver through the extensive proliferation of metacestodes, so identification of CE accompanied by HCC was more difficult in many clinical settings. Treatment principles were closely associated with the comprehensive classification of concomitant HCC and hydatid cyst [12]. According to Romic classification proposal, five co-existing cases were classified as type 3a HCC/CE lesion, four cases as type 1b HCC/CE lesion, two cases as type 4a HCC/CE lesion, one case as type 3b mHCC/CE lesion and one case as type 4b mHCC/CE lesion. In four cases, subtotal peri-cystectomy and hepatectomy were performed. However, only one patient received chemotherapy and conservative supporting care was considered as appropriate treating method for other patients, which was largely in line with the previous recommendations.

Echinococcosis with tumor is an extremely rare clinical scenario, which is closely associated with patients diminished quality of life and significant morbidity. Recent studies in Europe and central Asia have shown that there may exist a connection between hydatids and tumors [18–20]. Although great efforts have been made, most of the studies are animal experiments or basic researches. Thus, whether hydatid has anti-tumor effect is still not absolutely clear. Various research groups have reported that there may exist negative correlation between hydatid infection and cancer progression. Researchers discovered many antigenic similarities between e*chinococcus granulosus* and some malignant tumors through testing patients serum antigens [21–23]. Besides, based on the above evidence, Van Knapen F et al. have also put forward the hypothesis that echinococcus infection could suppress tumor growth [24]. Then, researchers found massive O-glycan antigen Tn in CE patients serum, which was also highly expressed in the serum of cancer patients. Ex-vivo studies have also demonstrated that hydatid cyst protoscolices could inhibit proliferation of WEHI-164 fibrosarcoma and baby hamster kidney fibroblasts [25]. Moreover, it was verified by animal studies that CE patients serum had an anti-tumor activity on the growth of non-small cell lung cancer and that mucin-like antigens in the hydatid cystic fluid could promote the proliferation of natural killer (NK) cells in human body to further kill cancer cells [16, 26]. Bangaru et al. came to the conclusion that hydatid infection could suppress colon cancer progression by treating colon cancer animals with *echinococcus granulosus*. Importantly, antigens from the protoscolices and hydatid cyst fluid are both able to decrease tumor size significantly in melanoma bearing animals [9], further suggesting that *echinococcus granulosu*s indeed share some common antigens with cancers cells, which is possible mechanistic anti-tumor activity of echinococcus in some malignant cases [27].

In this study, in order to primarily validate our hypothesis, we also reviewed HCC patients, which were matched according to the confounding factors (sex, age and tumor TMN stage). Interestingly, although there was no significant difference between etiological factors between two group patients, presence rate of virus infection and cirrhosis was slightly higher in patients with concomitant HCC and CE. Median survival time for HCC patients was 6 month (1–17). However, that was 8 month (3–90) for patients with both conditions, which was closely associated with tumor size, location, TMN stage and hydatid size, location, classification [28, 29] and our results were highly accorded with previous studies. Importantly, four patients underwent surgical intervention and median survival time for them was 17 month (3–68), which was significantly longer than reported average level by some researchers that the median survival time for advanced HCC patients was only 3–5 month [5].

However, our findings were in disagreement with a previous retrospective study which investigated the relationship between prior *echinococcus granulosus* infection and cancer development, an indication of possible cancer-causing risks of *echinococcus granulosus* infection [30]. In another retrospective study, it was shown that echinococcus infection may have a pro-cancerogenic effect through modulating the immune response. However, the study results were unable to determine the follow-up outcome for patients with hydatid disease and their initial studies were not adequate for detecting malignancy [31]. In addition, another was a case report that HCC was accidentally noted during the surgery of CE lesions [32]. In areas of endemicity, the annual CE incidence ranges from<1 to 200 per 100,000 and the mortality rate (2–4%) is lower but may increase considerably if inadequate care management is provided [33]. In our cohort, there were 13 patients who reported coexistent *echinococcus granulosus* infection and HCC out of the 3300 formerly infected subjects surveyed. Compared to the CE incidence, simultaneous occurrence of CE and HCC (0.39%) is extremely low. *Echinococcus granulosus* may exist within the human host concurrently with tumor cells and may induce the imbalance of immune system [34]. Thus, parasitic infection may induce tumourigenesis during the long-term coexisting period. However, relatively higher presence of virus infection and cirrhosis may be the chief culprit of ultimate HCC in the concomitant cases. In this regard, large number of clinical and animal studies are needed to clarify the specific roles of echinococcus infection in the cancer development.

## Conclusion

Taken together, it was suggested by our results that *echinococcus granulosus* may have anti-tumor activity towards HCC progression and significantly prolong HCC patients overall survival time. However, the specific mechanistic roles of *echinococcus granulosus* as a protective factor against cancer development have to be further confirmed by clinical and animal studies.

## Data Availability

All data supporting the conclusions were shown in this manuscript.

## Abbreviations

CE: Cystic echinococcus
CT: Computed tomography
DIGFA: Dot immunogold filtration assay
HCC: Hepatocellular carcinoma
MRI: Magnetic resonance imaging
NK: Nature killer
PMN: P: parasite mass in the liver, N: involvement of neighboring organs, M: distance metastasis
TNM: T: tumor, N: node, M: metastasis
US: Ultrasonography
WHO-IWGE: World Health Organization Informal Working Group on Echinococcosis
